# Association between psychotropic drug use and handgrip strength in older hospitalized patients

**DOI:** 10.1007/s41999-021-00511-6

**Published:** 2021-05-25

**Authors:** Miriam Kristine Sandvik, Leiv Otto Watne, Anniken Brugård, Marte Sofie Wang-Hansen, Hege Kersten

**Affiliations:** 1 Department of Psychiatry and Addiction, Telemark Hospital Trust, Skien, Norway; 2 Oslo Delirium Research Group, Department of Geriatric Medicine, Oslo University Hospital, Oslo, Norway; 3 Department of Medicine, Drammen Hospital, Vestre Viken Trust, Drammen, Norway; 4 Institute of Clinical Medicine, University of Oslo, Oslo, Norway; 5 Vestfold Hospital Trust, Tønsberg, Norway; 6 Department of Research, Telemark Hospital Trust, Skien, Norway; 7 Department of Pharmaceutical Bioscience, School of Pharmacy, University of Oslo, Oslo, Norway

**Keywords:** Psychotropic drugs, Frailty, Handgrip strength, Potentially inappropriate drugs

## Abstract

**Purpose:**

Handgrip strength is an indicator of frailty and longevity in older adults. The association between psychotropic drug use and handgrip strength in older hospitalized patients was investigated in this study.

**Methods:**

A total of 488 patients were included in this retrospective cross-sectional study, 333 women and 155 men, from two different cohorts of older (mean age 84 years) hospitalized in-patients. We used multiple linear regression models to explore the association between psychotropic drug use and handgrip strength. We adjusted for factors known to affect handgrip strength: Age, gender, body mass index (BMI) and comorbidity (Charlson comorbidity index).

**Results:**

Both unadjusted and adjusted analyses showed that psychotropic drug use was associated with handgrip strength (*β* = − 0.183, *p* < 0.0001). The relationship was of a linear character, with no clear threshold value, but with the greatest reduction in handgrip strength between zero and two psychotropic drugs.

**Conclusion:**

An increasing number of psychotropic drugs were significantly associated with reduced handgrip strength in a linearly pattern. Hence, it is timely to question the guided threshold value of avoidance of three or more psychotropic drugs in older people. Psychotropic drug use should be kept as low as possible in treatment of older patients.

**Supplementary Information:**

The online version contains supplementary material available at 10.1007/s41999-021-00511-6.

## Key summary points

**Aim:**

To investigate the association between psychotropic drug use and handgrip strength in older hospitalized patients.

**Findings:**

Psychotropic drug use was linearly associated with handgrip strength, with the greatest reduction in handgrip strength between zero and two psychotropic drugs.

**Message:**

Psychotropic drug use should be kept as low as possible in treatment of older patients.

**Supplementary Information:**

The online version contains supplementary material available at 10.1007/s41999-021-00511-6.

## Introduction

The use of psychotropic drugs is common among older people, although use of such drugs in the older persons is related to high risk of adverse effects, such as reduced cognitive functions, muscular weakness, tiredness, risk of falling and hip fractures [1, 2]. Psychotropic drugs include antipsychotics, antidepressants, anxiolytics and hypnotics, and many studies also include centrally acting analgesics [3]. Psychotropic drugs constituted approximately 60% of all potentially harmful drug prescriptions to home-dwelling patients over 70 years old in a previous Norwegian study, and 5% of those included used three or more psychotropic drugs at the same time [3]. Nursing home patients use an even higher amount of psychotropic drugs [4, 5], and there seem to be substantial variation between comparable nursing homes [6]. The use of psychotropic drugs in nursing homes has been increasing over the last decades, although recent studies indicate a decline in the use of antipsychotics [4, 7–10]. A recent study found that more than two-thirds of nursing home patients with dementia received at least one psychotropic drug [11]. Several studies find that women use more psychotropic drugs than men [3, 4].

Older patients are more prone to central adverse effects of psychotropic polypharmacy due to age-related pharmacokinetic and pharmacodynamics changes, such as increased permeability across the blood–brain-barrier, reduced metabolism, reduced kidney function and increased accumulation in fatty tissue, etc. [12]. Psychotropic drugs are often involved in drug-related problems caused by drug–drug interactions, drug–disease interactions and increased serum concentrations [12, 13]. Several criteria have been developed as recommendations to avoid potentially harmful drug use in older persons [14, 15]. These criteria all define use of specific psychotropic drugs, high doses of such drugs and combination of three or more centrally acting analgesics and/or psychotropics as inappropriate in older persons [5, 14, 15]. Some studies have found that concomitant use of several psychotropic drugs and/or opioids was significantly associated with adverse effects, such as reduced handgrip strength [16] and fractures [17, 18]. Despite that potentially inappropriate psychotropic drugs cause serious adverse drug reactions and acute hospitalization in vulnerable older patients, these drugs are still commonly in use [19–21]. Most guidelines recommend avoidance of three or more potentially harmful CNS-active drugs in older patients, but there is a need to evaluate whether this is a rational and useful threshold.

Handgrip strength has been shown to be a good indicator of general health and frailty in the older persons, and an independent predictor of death [22–25]. Weakness and risk of falling are among the main harmful outcomes of polypharmacy and use of potentially inappropriate medications (PIMs) [26, 27], and handgrip strength is often used as a measure of weakness [26]. Therefore, we wanted to explore the association between the use of psychotropics and opioids and handgrip strength in frail hospitalized older patients and evaluate whether a threshold of three potentially harmful central nervous system (CNS)-active drugs is rational and clinically useful [14].

## Methods

### Aim

To investigate the association between psychotropic drug use and handgrip strength in older hospitalized patients.

### Study population

Our study population was obtained from two patient cohorts: (1) 332 older patients acutely admitted with a hip fracture to Oslo University Hospital, Ullevål, in the period 2009–2012. The population is previously described in details [28]. (2) 232 multi-morbid patients ≥ 75 years old acutely admitted to the medical ward at Vestfold Hospital Trust in 2012. The most common main diagnoses in the Vestfold cohort at admission were dehydration, pneumonia, urinary infections, respiratory failure and acute renal failure. The population is described in more detail earlier [29].

304 Oslo patients and 184 Vestfold patients had registered handgrip strength measures and were included in the present analyses. Seventy-six patients did not measure handgrip strength. To evaluate whether omitting these patients introduced a selection bias to our study, we investigated differences between patients who did not measure handgrip strength, patients who had zero handgrip strength and patients who had handgrip strength above zero. The patients who did not measure handgrip strength were more similar to the patients with handgrip strength of zero (they were older and used a higher total number of drugs) than to patients with handgrip strength above zero.

The study was approved by the Regional Ethics Committee (case number 25754). The participants consented to participation in the study, and patient information is included in the approval from the Regional Ethics Committee.

### Variables

#### Psychotropic drugs

Prescribed drugs were registered with ATC codes and daily doses upon admission. Only drugs used regularly at the time of admission were included in the analyses. The updated 2015 version of Beers’ criteria for potentially inappropriate medication use in older adults advise to avoid combining three or more CNS-active drugs including antipsychotics; benzodiazepines; non-benzodiazepine hypnotics; tricyclic antidepressants (TCAs); selective serotonin reuptake inhibitors (SSRIs); and opioids [14]. Accordingly, we defined psychotropic drugs as antipsychotics, antidepressants, anxiolytics and sedatives and opioids.

#### Outcome variable

Handgrip strength was measured within the first three days of the hospital stay. For the Oslo cohort, handgrip strength was measured both pre- and postoperatively, and the highest value was used. A Jamar dynamometer was used in the assessments. Patients who were able to get out of bed performed the test sitting on a chair. Bedridden patients were assessed in a sitting position with the backrest elevated, with shoulders and arms held against their body in a naturally—rotated position, elbows bent at 90°, forearms in a neutral position, wrist between 0 and 30° dorsiflexion and between 0 and 15° ulnar deviation. The patients were instructed to squeeze the handle as forcefully as possible for 5 s. Three attempts were given, and results from the strongest hand were included in the analyses (measurements in kg). We used the threshold values identified in a large study conducted to determine age-dependent normative values for normal and reduced handgrip strength. The study found < 20 kg for women and < 30 kg for men to be useful threshold values to identify persons with mobility limitations [30], and these threshold values have later been recommended by the European Working Group on Sarcopenia in Older People (EWGSOP) [31].

#### Covariates

Information about medical diagnoses and morbidity was collected upon admission and during the acute stay in both cohorts. Comorbidity was assessed by the Charlson comorbidity index (CCI) [32]. Weight and height were registered at admission, and body mass index (BMI) calculated. In the Oslo cohort, information about pre-admission social and cognitive function was collected by interviewing proxies. Activity of daily living was rated by Barthel’s activities of daily living index (BADL) [33] and cognitive function was assessed by informant questionnaire on cognitive decline in the elderly (IQCODE) [34]. In the Vestfold cohort, cognitive function was registered using the Norwegian version of mini mental state examination (MMSE-NR3) [35, 36]. Since different assessments of cognitive function were used in the two cohorts, we did not include a specific measure of cognitive function as a covariate in the regression analyses. However, cognitive impairments and dementia are included and weighted in in the comorbidity index (CCI) and hence adjusted for in the multiple linear regression model.

### Statistical analyses

The study was conducted as a retrospective cross-sectional study. The patient cohorts were analysed separately before merging into one cohort. We used ANOVA to assess differences between groups for continuous variables. Pearson´s chi-square tests were used to assess categorical variables. Drug use and comorbidity variables were not normally distributed, and therefore, Spearman’s rank correlation coefficient was used to analyse correlations between variables. We used multiple linear regression models to assess whether psychotropic drug use was a predictor of handgrip strength. We conducted unadjusted and adjusted analyses. In the adjusted analyses, we adjusted for factors known to affect handgrip strength: age, gender, body mass index (BMI) and comorbidity [37]. Because of some significant differences between the two patient cohorts, we also adjusted for patient cohort. We tested collinearity between the independent factors using variation inflation factor (VIF), and VIF < 5 was considered not to interfere with the goodness-of-fit of the model. IBM SPSS Statistics version 23 was used for all statistical analyses.

## Results

A total of 564 patients were included in the study. Sixty-five of these patients (12%) had psychiatric diagnoses, most commonly depression and anxiety disorders (42 patients). Fifteen patients had substance abuse. Many patients had chronic somatic diseases, most commonly ischemic heart disease (39%), hypertension (26%), arrhythmias (23%), chronic obstructive pulmonary disease (18%) and diabetes mellitus (16%). Fifty per cent of the hip fracture patients had delirium during the hospital stay, while 30% were diagnosed with delirium in the multi-morbid Vestfold cohort.

Handgrip strength measures were registered for a total of 488 patients, 333 women and 155 men, and these patients were included in the further analyses.

Mean length of the hospital stay was 9.5 days (SD = 6.9 days). Mean age was 84 years. Approximately half of the patients had cognitive impairment, and half of the patients (Oslo cohort) had ADL impairment. Barthel’s ADL index was highly correlated to handgrip strength (Spearman’s correlation coefficient 0.499, *p* < 0.0001). Characteristics of the patient cohorts and the merged study population are shown in Table 1. According to the EWGSOP-recommended threshold values, the handgrip strength was reduced in the whole study population with a mean handgrip strength of 14.8 (SD = 7.9) kg for the women and 23.8 (SD = 11.3) kg for the men. In the merged population, the mean number of medications used regularly was 5.7 (SD = 3.7). Two-hundred-and-thirty-three patients (48%) used at least one psychotropic drug at admission, 100 (20%) patients used at least two psychotropic drugs and 45 patients (9.2%) used three or more psychotropic drugs. Excluding psychotropic drugs, mean number of drugs used regularly was 4.9 (SD = 3.5). The distribution of psychotropic drugs is shown in Table 2. Antidepressants were the most common psychotropic drug, used by 95 patients (19%).

**Table 1:** Characteristics

Characteristics						
	Total study population		Vestfold cohort (medical causes)		Oslo cohort (hip fracture)	
	Valid number of patients (*n*)	Mean (SD) or number (%)	Valid number of patients (*n*)	Mean (SD) or number (%)	Valid number of patients (*n*)	Mean (SD) or number (%)
Total		488 (100)		184 (100)		304 (100)
Women	488	333 (68.2)	184	108 (59)	304	225 (74)*
Age (years)	488	83.8 (8.3)	184	86.0 (8.3)	304	82.5 (9.2)*
BMI	396	24.1 (4.4)	169	23.7 (4.5)	227	24.4 (4.3)
Length of stay (days)	488	9.5 (6.9)	184	6.9 (4.9)	304	11.1 (8.0)*
Hand grip strength dominant hand						
All	488	17.7 (10.0)	184	15.3 (8.5)	304	19.1 (10.6)*
Men	155	23.8 (11.3)	76	21.5 (8.7)	79	26.1 (13.0)**
Men < threshold (30 kg)^a^	155	93 (60)	76	60 (79)	79	33 (42)*
Women	333	14.8 (7.9)	108	10.9 (4.8)	225	16.7 (8.4)*
Women < threshold (20 kg)^a^	333	188 (56)	108	91 (84)	225	97 (43)*
Cognitive function						
IQCODE^b^					296	3.8 (0.78)
MMSE^c^			180	22.8 (5.3)		
Total CHARLSON comorbidity index	488	1.1 (1.5)	184	1.0 (1.7)	304	1.2 (1.4)
Barthel ADL index^d^					302	16.7 (4.0)
ADL impairment (ADL 18 or lower)					302	163 (54%)
Total number of drugs	488	5.7 (3.7)	184	7.7 (3.7)	304	4.5 (3.2)*
Total number of drugs without psychotropics	488	4.9 (3.5)	184	7.0 (3.5)	304	3.6 (2.7)*

*Significant group difference with *p* < 0.0001

**Significant group difference with *p* = 0.01

^a^According to EWGSOP age and BMI appropriate threshold recommendations

^b^Informant questionnaire on cognitive decline in the elderly

^c^Mini mental state examination NR-3

^d^Barthel’s activities of daily living index

**Table 2:** Psychotropic drug use

	Total study population (*n* = 488) (%)	Vestfold cohort (medical causes) (*n* = 184) (%)	Oslo cohort (hip fracture) (*n* = 304) (%)	*p* value (Pearson chi-square test)
Psychotropic drugs	Frequency	Frequency	Frequency	
Zero	255 (52)	95 (52)	160 (53)	
At least one	233 (48)	89 (48)	144 (47)	0.83
Two or more	100 (20)	30 (16)	70 (23)	0.20
One or more antidepressants (N06A)	95 (19)	31 (17)	64 (21)	0.26
One or more antipsychotics (N05A)	41 (8.4)	9 (4.9)	32 (11)	0.030
One or more opiates (N02A)	74 (15)	27 (15)	47 (16)	0.81
One or more anxiolytics (N05B)	59 (12)	16 (8.7)	43 (14)	0.074
One or more hypnotics (N05CD/N05CF)	95 (19)	38 (21)	57 (19)	0.61

There were some differences between the two patient cohorts with regard to characteristics as shown in Table 1 and drug use as shown in Table 2. The Oslo patients with hip fracture were younger than the multi-morbid Vestfold patients, and a higher percentage were women. The Oslo patients had longer hospital stays. Comorbidity was similar in the two cohorts, but the Oslo patients used fewer drugs in total. The Oslo patients also had higher handgrip strength than the Vestfold patients, both overall and stratified for sex. Psychotropic drug use was fairly similar in both patient cohorts, with the exception of more use of antipsychotics in the Oslo cohort (Table 2).

First, we looked at psychotropic drug use as a dichotomous variable and found that handgrip strength was significantly lower (13.9 kg (SD = 8.6 kg) vs. 18.1 kg (SD = 10.1 kg), *p* = 0.007) in patients that used three or more psychotropic drugs compared to the rest of the study population. The same group had higher comorbidity, at a significant level only in the Oslo cohort (Oslo: total Charlson index 1.6 (SD = 1.8) vs. 1.1 (SD = 1.3), *p* = 0.029, Vestfold: total Charlson index 1.4 (SD = 1.4) vs. 1.0 (SD = 1.6), *p* = 0.48). Total number of drugs were significantly higher in the group who used three or more psychotropic drugs (8.3 (SD = 3.5) vs. 5.5 (SD = 3.6), *p* =  < 0.001), but was not significantly different between the groups when excluding psychotropic drugs from total number of drugs. In regression analyses, regular use of three or more psychotropic drugs was a significant predictor of reduced handgrip strength in both unadjusted (*β* = − 0.121, *p* = 0.007) and adjusted analyses (*β* = − 0.124, *p* = 0.002). Analyzing the two study groups separately showed the same trends with *β* = − 0,104, *p* = 0,087 in the Vestfold cohort and *β* = − 0.199, *p* < 0.0001 in the Oslo cohort.

**Fig. 1:**
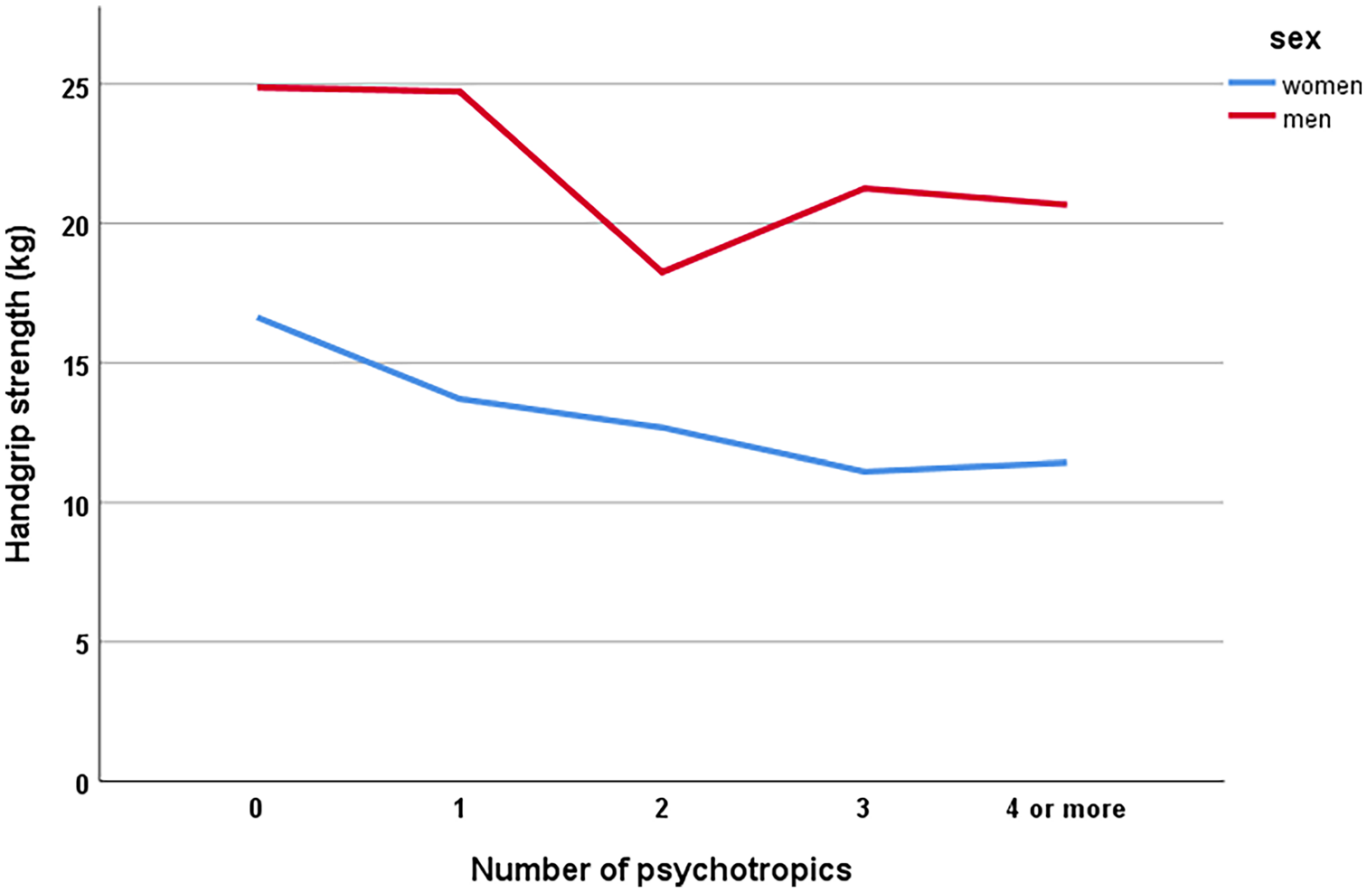
Handgrip strength and number of psychotropic drugs

Second, we looked at psychotropic drug use as a linear variable. Because of a limited number of patients using more than four psychotropic drugs, (only two patients used more than five; eight patients used five drugs; and seven patients used four) we merged these into one group. Multiple regression analyses showed that an increasing number of psychotropic drugs was associated with a reduction in handgrip strength in a linearly pattern, in both unadjusted and adjusted analyses (Table 3, Fig. 1). Analysed for sex separately, adjusted regression analyses showed similar results (women: *β* = − 0.236, *p* < 0.0001, men: *β* = − 0.190, *p* = 0.014). Sub-analyses of each drug class was limited by small group sizes, but antidepressants, antipsychotics and anxiolytics were the drug classes most strongly associated with reduced handgrip strength (antidepressants: *β* = − 0.126, *p* = 0.001, antipsychotics: *β* = − 0.185, *p* < 0.0001, anxiolytics: *β* = − 0.078, *p* = 0.042 in adjusted analyses).

**Table 3:** Predictors of handgrip strength

Predictors of handgrip strength	Unadjusted		Adjusted	
	*β*	*p*	*β*	*p*
Age	− 0.397	< 0.0001	− 0.274	< 0.0001
BMI	0.227	0.036	0.189	< 0.0001
Male sex	0.420	< 0.0001	0.463	< 0.0001
Total CHARLSON comorbidity index	− 0.008	0.859	− 0.054	0.151
Admission group (medical causes = 1, hip fracture = 0)	0.185	< 0.0001	0.239	< 0.0001
Psychotropic drugs as a linear variable	− 0.202	< 0.0001	− 0.183	< 0.0001

## Discussion

In this study of older hospitalized patients, we found that use of psychotropic drugs was significantly associated with reduced handgrip strength. The greatest reduction in handgrip strength was seen between zero and one psychotropic drug, and between one and two drugs, and the association between psychotropics and handgrip strength seemed to be linear (Fig. 1). Hence, our results do not support the commonly used cut-off of three or more psychotropic drugs as inappropriate [14], but rather indicate that the psychotropic drug use should be kept as low as possible in treatment of older patients.

It is noteworthy that almost half of our patients used one or more psychotropic drugs, a much larger proportion that the 12% with psychiatric diagnoses. Psychiatric diagnoses are probably under-reported in our data, as psychiatric diagnoses are not always registered when the main reason for admission is somatic. Nevertheless, the substantial use of psychotropic drugs, such as antipsychotics (8%) and anxiolytic/hypnotic drugs (31%), indicates a more liberal prescribing practice to older patients than recommended by existing guidelines. Frailty is an important predictor of prognosis in older patients, and associated with higher mortality, longer hospital stays and more complications [38, 39]. Handgrip strength is an indicator of frailty and a predictor of mortality for older patients. Moreover, ADL function was strongly correlated to handgrip strength in our study, and highlights handgrip strength as an important factor for daily life functioning in older persons. Our findings of reduced handgrip strength associated with psychotropic drug use emphasize the importance of avoiding unnecessary use of psychotropic drugs, and raises the question whether current guidelines is strict enough.

The discussion on polypharmacy as a concept has in the recent years questioned the clinical value of the common use of a cut-off number of five drugs to define polypharmacy, and some studies have demonstrated a linear relationship between the number of drugs used and drug-related problems [40–42]. Accordingly, our results demonstrate the same linear relationship between increasing number of psychotropics and reduced handgrip strength. Hence, the results do not support a cut-off number of three psychotropic drugs, but rather indicate that if a threshold number should be used in treatment recommendations, it would be advisable to avoid the combination of two or more potentially harmful CNS-active drugs.

The possible mechanisms to explain the impact of psychotropic drugs on handgrip strength are complex. Psychotropic drugs have a range of well-known side effects, such as changes in appetite, dizziness, drowsiness, fatigue and sleep disturbances, and such side effects may be more frequent and more severe in older persons due to age-related pharmacokinetic and pharmacodynamics changes [12]. Additive and synergistic effects and less dosage control due to pharmacokinetic drug interactions increase strongly with increasing number of drugs [43, 44]. Moreover, age-related changes in body composition and physical activity may affect muscle mass and handgrip strength [45]. Several psychotropic drugs, such as benzodiazepines, are muscle relaxants, and associated with increased risk of falling [46], and there is a consistent association between use of most classes of psychotropic drugs and risk of falling [1].

In this study, we have pooled two different cohorts of older hospitalized patients; one group admitted for medical conditions and one group due to hip fracture. Although the cohorts had some significant diversities, the association between psychotropic drug use and handgrip strength showed similar trends in both groups. Hence, we consider the heterogeneity in the study sample a strength that increases the generalizability, as we have included patients with a wider variety of characteristics than in most similar studies. Moreover, the prescription guidelines in question encompass both patient groups. The sample size in each patient cohort was a limiting factor in this study, and despite combining the two cohorts, a larger sample size is needed to give more precise estimates in analyses stratified on sex. Grouping together six differently acting types of drugs into the common variable “psychotropic drugs” is also a limitation, but necessitated due to the small sample size in each drug class. Although the results from the sub-analyses of drug classes therefore need to be interpreted with caution, we found that antidepressants, antipsychotics and anxiolytics were the drug classes most strongly associated with reduced handgrip strength.

Furthermore, our study population consisted of frail and hospitalized patients, with acute illness or hip fracture. They had a high degree of comorbidity and cognitive impairment, the latter probably affected in some of the patients by delirium caused by acute illness/injury. Adjusted for patient cohort, and despite the many present frailty factors affecting handgrip strength, we found a linear relationship between psychotropic drug use and handgrip strength. However, the association should be further investigated in a population of healthier and more stable patients. Seventy-six patients did not measure handgrip strength and were not included in further analyses. It is probable that many of these patients did not measure handgrip strength because they were too frail or did not understand the instructions, and excluding them introduces some selection bias. However, the patients without handgrip strength assessments showed similar characteristics as the patients with handgrip strength of zero and might have resulted in an underestimation of the actual association between handgrip strength and psychotropic drugs.

Psychotropic drug use was a significant predictor of frailty in older hospitalized patients, measured by handgrip strength. As there was a linear relationship between an increasing number of psychotropic drugs and reduced handgrip strength, this study gives reason to question current guidelines that advise against concurrent use of three or more psychotropic drugs in older people. Rather, our findings indicate that physicians should thoroughly question the need for each added psychotropic drug in older patients, and avoid such drug use if possible.

## Supplementary Information

Below is the link to the electronic supplementary material.

Supplementary file1 (DOCX 17 kb)

## Data Availability

An anonymized data file can be provided upon request.

## Abbreviations

PIMs: Potentially inappropriate medications
CNS: Central nervous system
TCAs: Tricyclic antidepressants
SSRIs: Selective serotonin reuptake inhibitors
EWGSOP: European Working Group on Sarcopenia in Older People
CCI: The Charlson comorbidity index
BMI: Body mass index (BMI)
BADL: Barthel’s activities of daily living index (BADL)
IQCODE: Informant questionnaire on cognitive decline in the elderly
MMSE: Mini mental state examination

## Code availability

SPSS syntax can be provided upon request.

## References

[1] Leipzig RM, Cumming RG, Tinetti ME (1999). Drugs and falls in older people: a systematic review and meta-analysis: I psychotropic drugs. J Am Geriatr Soc.

[2] Waade RB (2017). Psychotropics and weak opioid analgesics in plasma samples of older hip fracture patients—detection frequencies and consistency with drug records. Br J Clin Pharmacol.

[3] Nyborg G, Straand J, Brekke M (2012). Inappropriate prescribing for the elderly–a modern epidemic?. Eur J Clin Pharmacol.

[4] Ruths S (2013). Trends in psychotropic drug prescribing in Norwegian nursing homes from 1997 to 2009: a comparison of six cohorts. Int J Geriatr Psychiatry.

[5] Rognstad S (2009). The Norwegian General Practice (NORGEP) criteria for assessing potentially inappropriate prescriptions to elderly patients: a modified Delphi study. Scand J Prim Health Care.

[6] Fog AF (2020). Variation between nursing homes in drug use and in drug-related problems. BMC Geriatr.

[7] Halvorsen KH, Selbaek G, Ruths S (2017). Trends in potentially inappropriate medication prescribing to nursing home patients: comparison of three cross-sectional studies. Pharmacoepidemiol Drug Saf.

[8] Nygaard HA (2004). Not less but different: psychotropic drug utilization trends in Norwegian nursing homes during a 12-year period. The Bergen District Nursing Home (BEDNURS) Study. Aging Clin Exp Res.

[9] Vasudev A (2015). Trends in psychotropic dispensing among older adults with dementia living in long-term care facilities: 2004–2013. Am J Geriatr Psychiatry.

[10] Helvik AS (2016). Severity of neuropsychiatric symptoms in nursing home residents. Dement Geriatr Cogn Dis Extra.

[11] Callegari E (2021). Does psychotropic drug prescription change in nursing home patients the first 6 months after admission?. J Am Med Dir Assoc.

[12] Klotz U (2009). Pharmacokinetics and drug metabolism in the elderly. Drug Metab Rev.

[13] Hermann M, Waade RB, Molden E (2015). Therapeutic drug monitoring of selective serotonin reuptake inhibitors in elderly patients. Ther Drug Monit.

[14] By the American Geriatrics Society Beers Criteria Update Expert P (2015). American Geriatrics Society 2015 updated beers criteria for potentially inappropriate medication use in older adults. J Am Geriatr Soc.

[15] O'Mahony D (2015). STOPP/START criteria for potentially inappropriate prescribing in older people: version 2. Age Ageing.

[16] Kersten H (2015). Clinical impact of potentially inappropriate medications during hospitalization of acutely ill older patients with multimorbidity. Scand J Prim Health Care.

[17] Nurminen J (2010). Psychotropic drugs and the risk of fractures in old age: a prospective population-based study. BMC Public Health.

[18] Hartikainen S (2005). Concomitant use of analgesics and psychotropics in home-dwelling elderly people-Kuopio 75 + study. Br J Clin Pharmacol.

[19] Hilmer SN, Gnjidic D, Abernethy DR (2012). Pharmacoepidemiology in the postmarketing assessment of the safety and efficacy of drugs in older adults. J Gerontol A Biol Sci Med Sci.

[20] Wang-Hansen MS (2019). Can screening tools for potentially inappropriate prescriptions in older adults prevent serious adverse drug events?. Eur J Clin Pharmacol.

[21] Romskaug R (2020). Effect of clinical geriatric assessments and collaborative medication reviews by geriatrician and family physician for improving health-related quality of life in home-dwelling older patients receiving polypharmacy: a cluster randomized clinical trial. JAMA Intern Med.

[22] Koopman JJ (2015). Handgrip strength, ageing and mortality in rural Africa. Age Ageing.

[23] Bohannon RW (2008). Hand-grip dynamometry predicts future outcomes in aging adults. J Geriatr Phys Ther.

[24] Sallinen J (2010). Hand-grip strength cut points to screen older persons at risk for mobility limitation. J Am Geriatr Soc.

[25] Morley JE (2013). Frailty consensus: a call to action. J Am Med Dir Assoc.

[26] Jensen LD (2014). Potentially inappropriate medication related to weakness in older acute medical patients. Int J Clin Pharm.

[27] Hilmer SN, Gnjidic D (2009). The effects of polypharmacy in older adults. Clin Pharmacol Ther.

[28] Watne LO (2014). The effect of a pre- and postoperative orthogeriatric service on cognitive function in patients with hip fracture: randomized controlled trial (Oslo orthogeriatric trial). BMC Med.

[29] Moen K (2018). Physical function of elderly patients with multimorbidity upon acute hospital admission versus 3 weeks post-discharge. Disabil Rehabil.

[30] Lauretani F (2003). Age-associated changes in skeletal muscles and their effect on mobility: an operational diagnosis of sarcopenia. J Appl Physiol.

[31] Cruz-Jentoft AJ (2010). Sarcopenia: European consensus on definition and diagnosis: report of the European working group on sarcopenia in older people. Age Ageing.

[32] Charlson ME (1987). A new method of classifying prognostic comorbidity in longitudinal studies: development and validation. J Chronic Dis.

[33] Wade DT (1992). Measurement in neurological rehabilitation. Curr Opin Neurol Neurosurg.

[34] Jorm AF (1994). A short form of the informant questionnaire on cognitive decline in the elderly (IQCODE): development and cross-validation. Psychol Med.

[35] Folstein MF, Folstein SE, McHugh PR (1975). “Mini-mental state”. A practical method for grading the cognitive state of patients for the clinician. J Psychiatr Res.

[36] Strobel C, Engedal K (2008) MMSE-NR. Norwegian revised mini mental state examination. Revised and expanded manual

[37] Massy-Westropp NM (2011). Hand grip strength: age and gender stratified normative data in a population-based study. BMC Res Notes.

[38] Gilbert T (2018). Development and validation of a Hospital Frailty Risk Score focusing on older people in acute care settings using electronic hospital records: an observational study. Lancet.

[39] van Vliet M, Huisman M, Deeg DJH (2017). Decreasing hospital length of stay: effects on daily functioning in older adults. J Am Geriatr Soc.

[40] Viktil KK (2007). Polypharmacy as commonly defined is an indicator of limited value in the assessment of drug-related problems. Br J Clin Pharmacol.

[41] Masnoon N (2017). What is polypharmacy? a systematic review of definitions. BMC Geriatr.

[42] Rambhade S (2012). A survey on polypharmacy and use of inappropriate medications. Toxicol Int.

[43] Gnjidic D (2012). Polypharmacy cutoff and outcomes: five or more medicines were used to identify community-dwelling older men at risk of different adverse outcomes. J Clin Epidemiol.

[44] Doan J (2013). Prevalence and risk of potential cytochrome P450-mediated drug-drug interactions in older hospitalized patients with polypharmacy. Ann Pharmacother.

[45] Konig M (2017). Polypharmacy as a risk factor for clinically relevant sarcopenia: results from the berlin aging study II. J Gerontol A Biol Sci Med Sci.

[46] Alvarez CA (2015). Association of skeletal muscle relaxers and antihistamines on mortality, hospitalizations, and emergency department visits in elderly patients: a nationwide retrospective cohort study. BMC Geriatr.

