# Supplementary material for: Association between psychotropic drug use and handgrip strength in older hospitalized patients
Source: Eur Geriatr Med. 2021 May 25;12(6):1213–20. doi: 10.1007/s41999-021-00511-6 (PMC8626357; doi:10.1007/s41999-021-00511-6)
Supplement: Supplementary file 1 — Supplementary file1 (DOCX 17 kb) [file 41999_2021_511_MOESM1_ESM.docx]

**Diagnoses**

| **Diagnoses** | **Oslo cohort** | **Vestfold cohort** | **All patients** | |
| --- | --- | --- | --- | --- |
|  | N | N | N | % |
| All patients | 332 | 232 | 564 | 100 |
| Psychiatric diagnoses | 51 | 14 | 65 | 11.5 |
| Substance abuse/addiction | 11 | 4 | 15 | 2.7 |
| Schizophrenia | 1 | 0 | 1 | 0.18 |
| Bipolar affective disease | 4 | 0 | 4 | 0.71 |
| Depression | 21 | 2 | 23 | 4.1 |
| Anxiety disorders | 11 | 8 | 19 | 3.4 |
| Other | 3 | 0 | 3 | 0.53 |
|  |  |  |  |  |
| Chronic somatic illness |  |  |  |  |
| Parkinson´s disease | 7 | 6 | 13 | 2.3 |
| Other neurological diseases | 29 | 9 | 38 | 6.7 |
| Hypertension | 133 | 112 | 145 | 25.7 |
| Arrhythmias | 61 | 71 | 132 | 23.4 |
| Ischemic heart disease | 117 | 103 | 220 | 39.0 |
| Heart failure | 28 | 55 | 82 | 14.5 |
| Cerebrovascular disease | 48 | 12 | 60 | 10.6 |
| Chronic obstructive pulmonary disease | 49 | 54 | 103 | 18.3 |
| Renal failure chronic | 17 | 66 | 83 | 14.7 |
| Osteoporosis | 45 | 12 | 67 | 11.9 |
| Hypothyreosis | 28 | 12 | 40 | 7.1 |
| Diabetes mellitus | 46 | 47 | 93 | 16.5 |
